# Plasma Metabolomics of Thrombectomy‐Treated Patients Highlights Metabolic Pathways Underlying Disease and Recovery

**DOI:** 10.1002/cns.71018

**Published:** 2026-07-09

**Authors:** Min Cai, Xiangyu Hou, Shilong Deng, Wenxin Shen, Dingzhi Gao, Xinyu Zhang, Siyuan Fan, Yongliang Xia, Yuanlin Ma, Ke Cheng

**Affiliations:** ^1^ Department of Nephropathy and Rheumatism The Affiliated Yongchuan Hospital of Chongqing Medical University General Practice School of Chongqing Medical University Chongqing China; ^2^ Department of Neurology The Affiliated Yongchuan Hospital of Chongqing Medical University, Chongqing Key Laboratory of Cerebrovascular Disease Research General Practice School of Chongqing Medical University Chongqing China; ^3^ Department of Neurology, Peking Union Medical College Hospital Chinese Academy of Medical Sciences and Peking Union Medical College Beijing China

**Keywords:** LC‐MS/MS, metabolomics, recanalization biomarkers, thrombosis

## Abstract

**Background and Aims:**

Thrombosis, characterized by pathological intravascular clot formation, underlies major clinical events such as cerebral infarction, myocardial infarction, and pulmonary embolism. However, metabolomic alterations associated with cerebral infarction, particularly those distinguishing patients with different clinical outcomes after recanalization, remain poorly characterized. This study aimed to provide an unbiased, systems‐level characterization of metabolic alterations in cerebral infarction thrombosis.

**Methods:**

We performed LC–MS/MS‐based untargeted plasma metabolomics in both positive and negative ion modes. The cohort included 24 patients with meaningful functional recovery after cerebral infarction thrombectomy (MFR), 21 patients with futile recanalization (FTR), and 20 healthy controls (CON). Global metabolomic profiles were analyzed using multivariate and univariate approaches, followed by KEGG pathway enrichment analysis.

**Results:**

Plasma metabolite profiles differed significantly among groups. Both MFR and FTR patients exhibited marked metabolic perturbations relative to controls, while the MFR group showed subtle but distinct metabolic patterns compared with FTR patients. Differential metabolites were enriched in pathways related to mitochondrial function, lipid signaling, amino acid metabolism, and inflammatory regulation, which are associated with endothelial function and thrombo‐inflammatory responses in cerebral infarction.

**Conclusion:**

This study identifies metabolic alterations associated with cerebral infarction thrombosis and clinical outcome status following recanalization. These findings highlight candidate metabolites and pathways that may inform future mechanistic studies and biomarker validation specific to cerebral infarction.

## Graphical abstract

Untargeted plasma metabolomics reveals distinct metabolic signatures distinguishing thrombosis patients with successful vs. failed recovery after recanalization. Altered pathways involving mitochondrial function, lipid metabolism, amino‐acid utilization, and inflammation highlight potential biomarkers and therapeutic targets for monitoring disease progression and improving post‐thrombectomy outcomes.

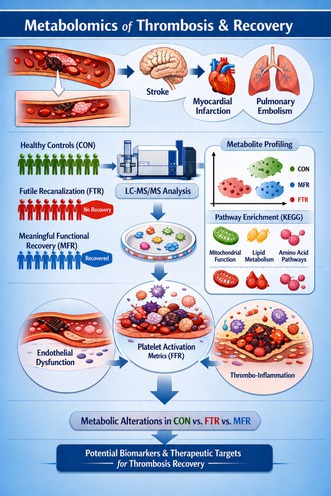

## 1 Introduction

Thrombosis arises from inappropriate blood clot formation within vessels, disrupting blood flow and causing profound clinical consequences. In the cerebrovascular system, arterial clots are the primary cause of ischemic stroke, also known as cerebral infarction, which is a leading cause of disability and death worldwide. While arterial or venous clots can trigger acute events such as myocardial infarction, ischemic stroke, deep vein thrombosis, and pulmonary embolism [1, 2], contributing to both emergency cardiovascular admissions and long‐term complications such as post‐thrombotic syndrome and persistent pulmonary hypertension [3], this study focuses specifically on metabolic alterations related to cerebral infarction. A wide spectrum of factors contributes to thrombus formation, including cardiometabolic disorders, lifestyle‐related risks, systemic inflammation, malignancy, and inherited prothrombotic traits. As populations age and non‐communicable diseases become more prevalent globally, the burden of thrombotic disorders continues to increase. Recent epidemiological analyses indicate that diseases driven by abnormal clot formation account for approximately one quarter of all deaths worldwide, highlighting their major public health impact [4, 5, 6].

Thrombosis treatment typically relies on anticoagulation, antiplatelet therapy, or pharmacological thrombolysis [7, 8], while severe cases require catheter‐based or surgical intervention [9]. Despite these therapeutic advances, monitoring the trajectory of recovery after treatment remains challenging. Conventional imaging and biomarkers provide only limited snapshots of vascular status and often fail to capture dynamic physiological changes during clot resolution and tissue repair. In this context, metabolomics offers a promising approach, as metabolites reflect real‐time biochemical activity and systemic responses to cerebral infarction, treatment, and recovery [10, 11]. However, comprehensive metabolite‐based frameworks for tracking recovery are still lacking. Developing such approaches could improve longitudinal monitoring, enable earlier detection of complications, and enhance understanding of the metabolic pathways that govern thrombotic disease progression.

Metabolomics is a powerful approach for characterizing the biochemical alterations underlying health and disease by profiling a broad array of small‐molecule metabolites, thereby providing mechanistic insights into physiological and pathological processes [12, 13]. In thrombosis research, metabolomics has uncovered important links between clot formation and diverse metabolic networks, including coagulation cascades, inflammatory signaling, lipid metabolism, oxidative stress responses, and endothelial dysfunction [11, 14, 15, 16], thereby enhancing our understanding of thrombus initiation and propagation. However, relatively few studies have explored how metabolic profiles evolve during postoperative or post‐interventional recovery, especially in patients with cerebral infarction [17]. Thrombus resolution and vascular healing involve coordinated processes, such as dampening inflammation, restoring vascular integrity, rebalancing hemostasis, and tissue remodeling, which are likely reflected in the circulating metabolome [17, 18]. Yet, the metabolic profiles associated with functional recovery after cerebral infarction remain poorly defined.

Non‐targeted metabolomics provides a powerful strategy for addressing this clinical gap by surveying thousands of metabolites simultaneously without predefined targets, enabling an unbiased, system‐wide view of metabolic dynamics during cerebral infarction recovery [13, 19]. Such comprehensive profiling increases the likelihood of detecting previously unrecognized biomarkers and uncovering novel pathways involved in vascular healing and systemic stabilization. Unlike targeted assays, which focus on a limited set of molecules, non‐targeted methods capture the full complexity of recovery‐related metabolic shifts [12]. Applying this strategy to postoperative thrombosis patients with cerebral infarction holds substantial promise for identifying indicators that reflect real‐time physiological status, support individualized clinical assessment, and enhance monitoring. Defining metabolomic profiles of cerebral infarction recovery may ultimately facilitate earlier detection of adverse outcomes and deepen understanding of the biological processes underlying successful restoration.

In the present study, we conducted a non‐targeted, comprehensive analysis of plasma metabolites in healthy controls (CON), futile recanalization (FTR) patients, and meaningful functional recovery (MFR) patients following cerebral infarction thrombectomy. Comprehensive metabolomic profiling captures both subtle and large‐scale metabolic changes, facilitating the identification of candidate biomarkers and potential mechanistic intermediates. In the context of postoperative or disease recovery, non‐targeted metabolomics can reveal coordinated metabolic profiles reflecting inflammation resolution, tissue remodeling, and restoration of homeostasis. These insights provide a framework for understanding metabolic differences associated with clinical outcomes after cerebral infarction and may guide the development of more precise monitoring strategies. By employing liquid chromatography–tandem mass spectrometry (LC–MS/MS) to characterize metabolite differences among CON, FTR, and MFR groups, this study provides a resource for subsequent investigations into treatment response and recovery monitoring following recanalization for cerebral infarction, thereby expanding current perspectives in this field.

## 2 Materials and Methods

### 2.1 Participants

Three groups of subjects were included in this study: CON, MFR, and FTR. The CON group consisted of 20 healthy volunteers without a history of stroke, thrombosis, coronary heart disease, or diabetes mellitus. None of these individuals were using anticoagulant medications at the time of enrollment. All patients in the MFR and FTR groups had arterial embolic diseases and underwent mechanical thrombectomy (MT) to remove the thrombus, with some patients also receiving combined pharmacologic thrombolysis. The MFR group comprised 24 patients who achieved successful reperfusion with favorable outcomes and functional recovery following thrombectomy. These patients completed follow‐up within six months after the operation, with clinical symptoms significantly improved and no active neurological or performance issues. The FTR group included 21 patients with poor outcomes who failed to recover after recanalization treatment (futile recanalization). These patients continued to show significant neurological dysfunction after surgery, with a 90‐day modified Rankin Scale (mRS) score > 2, indicating poor clinical function recovery.

To quantitatively distinguish MFR from FTR, a 90‐day modified Rankin Scale (mRS) score of 0–2 was used as the standard criterion for a good functional outcome [20, 21]. Although the 90‐day mRS is the primary outcome measure, early indicators, including discharge mRS (0–2) and 24‐h NIHSS scores (≤ 4), were used to further stratify patients and predict long‐term recovery. All participants were followed postoperatively for six months and were confirmed to be clinically stable without ongoing symptoms. Blood samples for LC–MS/MS analysis were collected within 24 h after thrombectomy.

Inclusion criteria were: Absence of previous stroke or thrombosis for controls; completed follow‐up within six months for patient groups; documented clinical status assessed via mRS and NIHSS scores. Exclusion criteria included individuals with active infections, autoimmune diseases, or other chronic health problems that could affect metabolic profiles.

### 2.2 Sample Collection and Processing

Plasma samples were obtained from participants after an overnight fast to minimize metabolic variability. Blood was collected into EDTA anticoagulant tubes and centrifuged at 2,000 × g for 10 min at 4°C, after which the plasma fraction was carefully separated for further analysis. Next, 300 μL of methanol/acetonitrile/water (2:2:1, v/v/v) was added to each sample, followed by vortexing for 30 s and ultrasonic extraction in an ice–water bath for 10 min. The mixture was then incubated at −20°C for 1 h to precipitate proteins. After incubation, samples were centrifuged at 13,000 rpm for 15 min at 4°C, and the supernatant was collected. The collected supernatant was transferred to a rotary evaporator and dried at 4°C. The residue was reconstituted in 100 μL of acetonitrile/water (7:3, v/v), followed by centrifugation at 13,000 rpm for 15 min at 4°C. The final supernatant was collected and stored at −80°C until analysis.

### 2.3 HPLC‐MS/MS Analysis

Metabolite profiling was performed using HPLC–MS/MS on a SCIEX ZenoTOF 7600 mass spectrometer (AB SCIEX, USA) coupled to an ExionLC UHPLC system (AB SCIEX, USA). Chromatographic separation was achieved on a Waters ACQUITY UPLC HSS T3 column (1.8 μm, 2.1 × 100 mm; Waters). A 2‐μL aliquot of each sample was injected, and analytes were separated using a 12‐min gradient. The mobile phase flow rate was maintained at 300 μL/min, and the column temperature was set at 40°C.

Mass spectrometric detection was performed using electrospray ionization in both positive and negative ion modes. Data were acquired using an information‐dependent acquisition (IDA) method, enabling simultaneous collection of full‐scan MS and MS/MS spectra. Mass data were recorded over an m/z range of 60–1,200 Da. The ion spray voltage was set to 5,500 V in positive mode and 4,500 V in negative mode, with the ion‐transfer capillary temperature set at 500°C. Curtain gas, nebulizer gas, and heater gas were set to 35, 45, and 45 arbitrary units, respectively. The collision energy was fixed at 35 V.

### 2.4 Metabolome Data Preprocessing

Raw MS data were converted to mzXML format, and MS/MS files were converted to MGF format using ProteoWizard (version 3.0.6150). Peak detection, integration, and retention time alignment were performed using the XCMS package in R (version 1.46.0). The resulting feature table was imported into MetDNA (http://metdna.zhulab.cn/) for metabolite annotation, with compound identities assigned based on matches to reference databases, including KEGG and HMDB. Annotation confidence was categorized from Level 1 (highest confidence) to Level 3 (putative identification).

Quality control (QC) performance was assessed by calculating the relative standard deviation (RSD) of QC features, with RSD values below 30% considered indicative of acceptable analytical stability. In addition, principal component analysis (PCA) of QC samples was performed to evaluate clustering behavior as an additional measure of analytical reproducibility.

### 2.5 Statistical Analysis

Both multivariate and univariate statistical analyses were performed to interrogate the metabolomic data. Unsupervised principal component analysis (PCA) was first conducted to assess overall sample distribution and inter‐group variability. Subsequently, supervised orthogonal partial least squares discriminant analysis (OPLS‐DA) was applied to evaluate group separation and identify metabolites contributing to between‐group differences. Metabolites with a variable importance in projection (VIP) score ≥ 1 were considered to contribute substantially to group discrimination. To assess model robustness and minimize overfitting, permutation testing (100 iterations) was performed.

Differential metabolites were identified based on combined multivariate and univariate criteria: VIP ≥ 1 from the OPLS‐DA model and statistical significance in univariate testing. For comparisons involving multiple tests, false discovery rate (FDR) correction was applied using the Benjamini–Hochberg procedure, and adjusted q‐values were used to determine significance. For analyses involving a limited number of predefined variables, unpaired two‐tailed Student's *t*‐tests were applied, with statistical significance defined as *p* < 0.05. Pearson correlation analysis was further conducted to evaluate associations among metabolites.

## 3 Results

### 3.1 Baseline Demographic Characteristics of the Study Cohort

A total of 65 adults were included in this study, comprising healthy controls (CON, *n* = 20) and two patient groups: Futile recanalization (FTR, *n* = 21) and meaningful functional recovery (MFR, *n* = 24). There were no significant differences in age or sex distribution between the control group and either patient group (Table 1). A modest but statistically significant difference in mean age was observed between the FTR and MFR groups (*p* = 0.03).

**TABLE 1:** Demographic characteristics of healthy controls and patient groups.

	CON	FTR	MFR	*p* value
Total number	20	21	24	—
Sex^a^ (female/male)	10/10	11/10	11/13	> 0.90
Age (year)				
Range	56–87	58–87	53–80	—
Average^b^ (mean ± SD)	69.80 ± 11.78	73.14 ± 8.55	66.00 ± 7.22	0.37 (CON vs. MFR)
				0.48 (CON vs. FTR)
				0.03 (MFR vs. FTR)

^a^ Statistical significance was calculated using the Chi‐square (and Fisher's exact) test.

^b^ Ordinary one‐way ANOVA with Tukey's post hoc test.

### 3.2 Untargeted Metabolomics Profiling of Plasma Samples

In LC–MS–based metabolomics, positive and negative ion modes represent two complementary ionization strategies, each preferentially detecting different classes of metabolites and generating distinct ion species [22, 23, 24]. To broaden metabolite coverage and enhance annotation confidence, data were acquired in both ionization modes.

**FIGURE 1:**
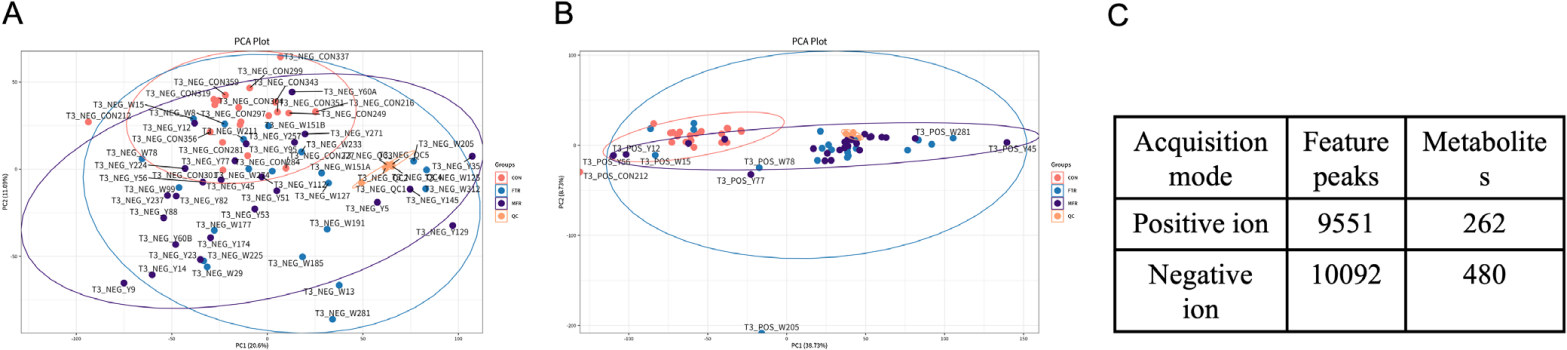
PCA score plots representing metabolic profiles of CON, FTR, and MFR subjects obtained by LC–MS in positive and negative ionization modes. (A) PCA score plot in negative‐ion mode (R^2^X(cum) = 0.507). (B) PCA score plot in positive‐ion mode (R^2^X(cum) = 0.546). The ellipse represents the 95% confidence interval. (C) Summary of chromatographic features and annotated metabolites in both ionization modes.

Principal component analysis (PCA) showed that the quality control (QC) samples, prepared by pooling equal volumes of all study samples, clustered tightly with minimal variability (Figure 1A,B). This tight clustering indicates excellent analytical stability and high overall data quality, supporting the reliability of the LC–MS/MS results. In positive‐ion mode, 10,092 chromatographic features were detected, of which 480 were confidently annotated (Figure 1C). In negative‐ion mode, 9551 features were detected, of which 262 were successfully annotated (Figure 1C). Metabolite annotation was performed using the MetDNA platform. Confidence in metabolite assignment was supported by an integrated evaluation of retention time, peak area, mass‐to‐charge ratio (m/z), and MS/MS fragmentation patterns. Most annotated metabolites met Level 1 identification criteria, reflecting a high degree of confidence in metabolite characterization.

### 3.3 CON, FTR, And MFR **Metabolomics** Differ on a Global Scale

**FIGURE 2:**
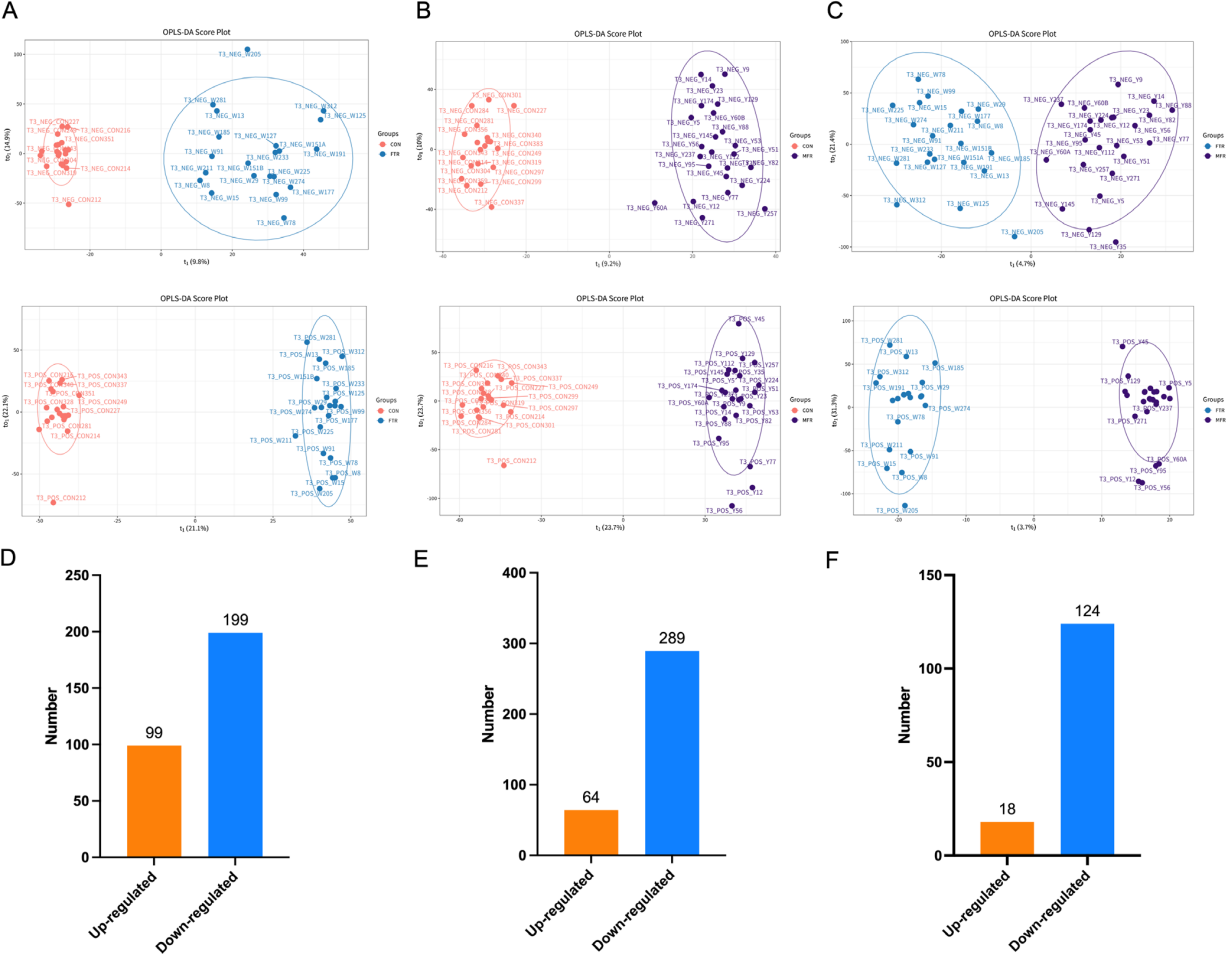
OPLS‐DA score plots showing separation between each pair of groups. (A) Comparison of overall metabolomic profiles between FTR and CON groups: Negative‐ion mode (top; R^2^X(cum) = 0.323, R^2^Y(cum) = 0.926, Q^2^(cum) = 0.603) and positive‐ion mode (bottom; R^2^X(cum) = 0.629, R^2^Y(cum) = 0.994, Q^2^(cum) = 0.787). (B) Comparison between MFR and CON groups: Negative‐ion mode (top; R^2^X(cum) = 0.467, R^2^Y(cum) = 0.976, Q^2^(cum) = 0.756) and positive‐ion mode (bottom; R^2^X(cum) = 0.586, R^2^Y(cum) = 0.989, Q^2^(cum) = 0.844). (C) Comparison between MFR and FTR groups: Negative‐ion mode (top; R^2^X(cum) = 0.344, R^2^Y(cum) = 0.869, Q^2^(cum) = 0.454) and positive‐ion mode (bottom; R^2^X(cum) = 0.621, R^2^Y(cum) = 0.987, Q^2^(cum) = 0.727). Ellipses represent the 95% confidence interval. (D–F) Number of differentially expressed metabolites for each pairwise comparison. (D) FTR vs. CON, (E) MFR vs. CON, (F) MFR vs. FTR.

To compare global metabolomic profiles among the CON, FTR, and MFR groups, we performed orthogonal partial least squares discriminant analysis (OPLS‐DA). Pairwise OPLS‐DA score plots demonstrated separation between groups (Figure 2A–C and Table S1), particularly in the positive‐ion mode, suggesting differences in metabolic profiles. In addition, samples from the FTR and MFR groups exhibited a more dispersed distribution than those from the CON group in both ionization modes (Figure 2A,B), indicating increased variability in metabolite composition within the patient groups.

Based on predefined criteria (VIP ≥ 1 and statistical significance in univariate analysis), 298 metabolites were identified as differentially expressed between the FTR and CON groups, including 99 upregulated and 199 downregulated metabolites in FTR (Figure 2D). In the MFR vs. CON comparison, 353 metabolites were differentially expressed, with 64 upregulated and 289 downregulated in MFR (Figure 2E). In contrast, differences between the FTR and MFR groups were comparatively modest, with 18 metabolites upregulated and 124 downregulated in MFR (Figure 2F).

### 3.4 Differential Metabolite Profiles and Group Comparisons

**FIGURE 3:**
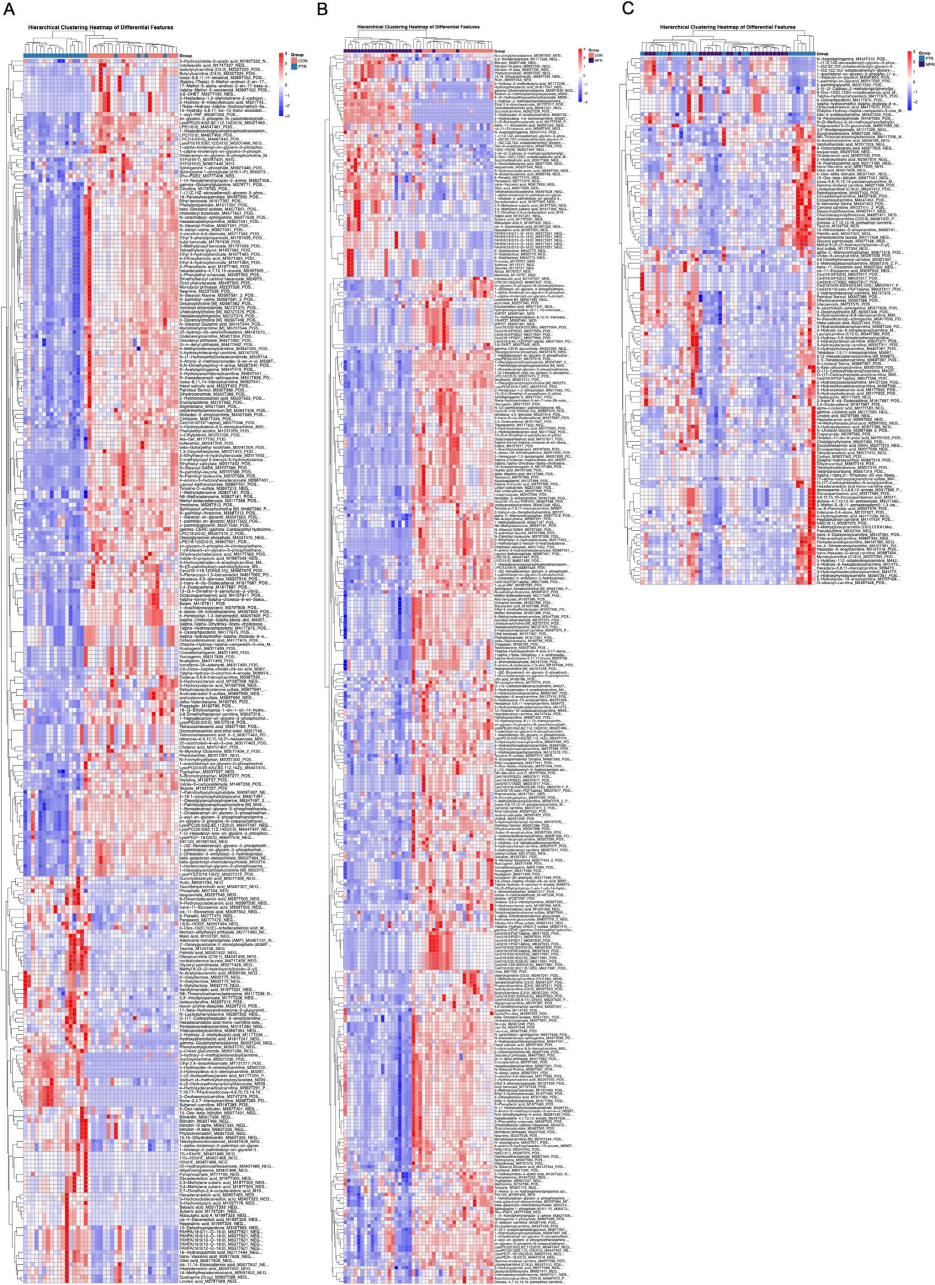
Heatmap of differentially expressed metabolites across group comparisons. (A) FTR vs. CON. (B) MFR vs. CON. (C) MFR vs. FTR. Hierarchical clustering illustrates group‐specific metabolite expression patterns. Red indicates higher metabolite abundance, and blue indicates lower abundance.

To visualize variation in metabolite abundance across groups, we generated a heatmap illustrating the expression patterns of key differentially expressed metabolites in the CON, FTR, and MFR groups (Table S2). Consistent with the multivariate analysis, both the FTR vs. CON and MFR vs. CON comparisons showed widespread differences in metabolite profiles, with a greater number of metabolites downregulated in the two recanalization groups relative to controls (Figure 3A,B). In contrast, the MFR vs. FTR comparison revealed comparatively modest differences (Figure 3C), indicating that the metabolic profiles of the two patient groups were more similar to each other than to those of healthy controls. The FTR vs. CON and MFR vs. CON comparisons highlight metabolic differences between patient groups and healthy baseline profiles, whereas the MFR vs. FTR comparison provides a direct assessment of metabolic variation between clinical outcome groups.

**FIGURE 4:**
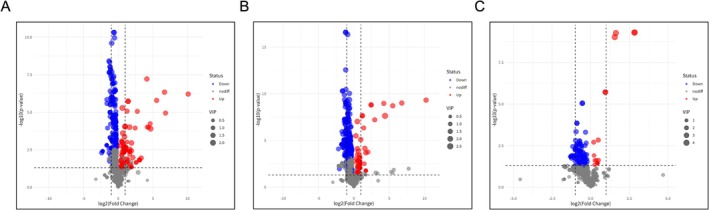
Volcano plots illustrating metabolite changes between groups. (A) FTR vs. CON. (B) MFR vs. CON. (C) MFR vs. FTR.

To further characterize the magnitude and statistical significance of these changes, we constructed volcano plots for each pairwise comparison, integrating fold change, *p*‐value, and variable importance in projection (VIP) (Figure 4A–C and Table S3). Across comparisons, upregulated metabolites generally exhibited larger fold changes than downregulated metabolites. Notably, VIP values were broadly comparable across metabolites, suggesting similar contributions to group discrimination.

Overall, these visualizations highlight distinct yet partially overlapping metabolic differences among groups. Further studies are needed to determine the potential diagnostic or prognostic relevance of these metabolites.

### 3.5 Metabolic Pathway and Function Analysis

To further interpret the observed metabolic alterations, KEGG pathway enrichment analysis was performed using metabolites with assigned KEGG identifiers in each pairwise comparison among the three groups (Table S4). Distinct sets of significantly enriched metabolic pathways were identified for each comparison.

**FIGURE 5:**
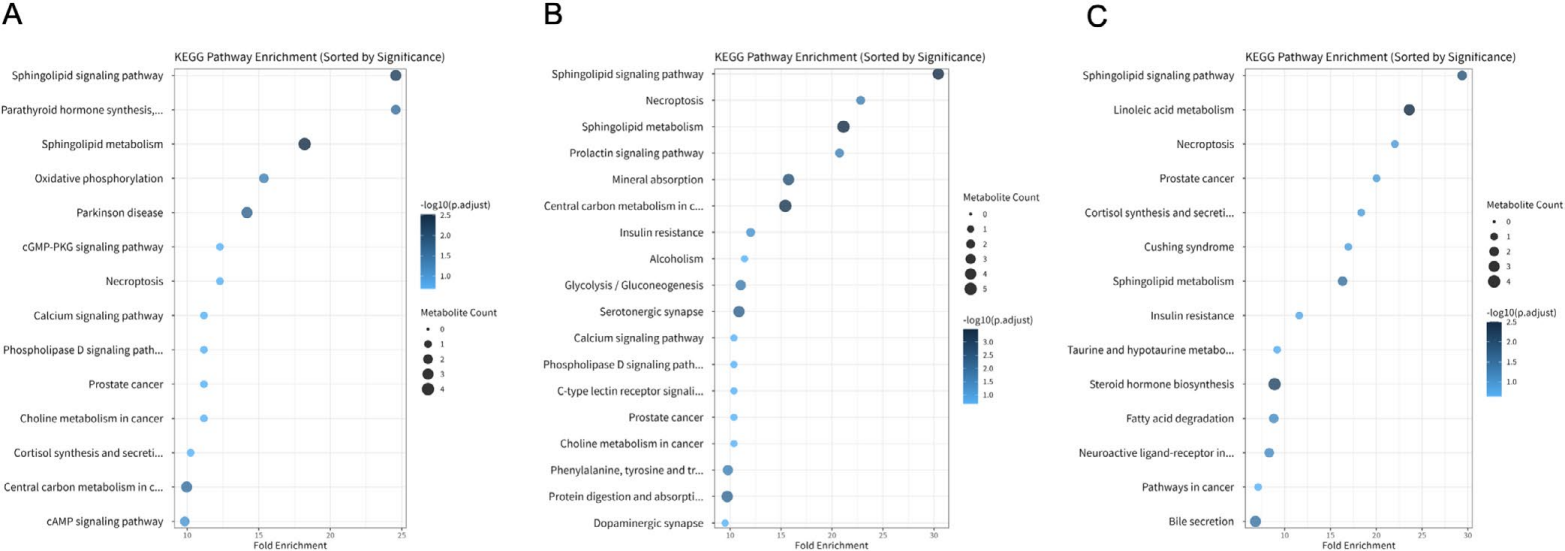
KEGG pathway enrichment analysis of significantly altered metabolites, showing the top enriched pathways ranked by fold enrichment and statistical significance. (A) FTR vs. CON. (B) MFR vs. CON. (C) MFR vs. FTR. Bubble size represents the number of metabolites mapped to each pathway, and color indicates enrichment (−log_10_ adjusted *p*‐value).

Compared with the CON group, the FTR group showed significant enrichment in pathways including sphingolipid signaling; parathyroid hormone synthesis, secretion, and action; sphingolipid metabolism; oxidative phosphorylation (OXPHOS); and pathways related to AMP, phosphate, and pyrophosphate metabolism in Parkinson's disease (PD), as well as central carbon metabolism in cancer (Figure 5A). In the MFR group, enrichment was observed not only in sphingolipid signaling, sphingolipid metabolism, and central carbon metabolism in cancer, but also in pathways related to glycolysis/gluconeogenesis; phenylalanine, tyrosine, and tryptophan biosynthesis; necroptosis; serotonergic synapse; prolactin signaling; protein digestion and absorption; and mineral absorption (Figure 5B). When comparing the MFR and FTR groups, five pathways were significantly enriched in the MFR group: Steroid hormone biosynthesis, linoleic acid metabolism, bile secretion, sphingolipid signaling, and sphingolipid metabolism (Figure 5C).

Taken together, pathways associated with sphingolipid signaling and metabolism were enriched in both the FTR and MFR groups, consistent with previously reported roles of sphingolipids in thrombosis‐related processes, including platelet activation, endothelial function, inflammation, and coagulation. Pathways uniquely enriched in the MFR group may reflect metabolic differences associated with recovery following recanalization. Further studies will be necessary to evaluate their potential as candidate biomarkers of treatment response or recovery.

## 4 Discussion

In the present study, we applied an untargeted LC–MS/MS‐based metabolomics approach to identify potential metabolic biomarkers in patients with cerebral infarction and to evaluate metabolic changes associated with recovery. Significant differences in plasma metabolite profiles were observed not only between FTR patients and healthy controls (Figures 3A and 4A), but also between the recovery group and healthy controls (Figures 3B and 4B). These alterations spanned multiple pathways and biological processes. Several of the most discriminative metabolites were further mapped onto the corresponding perturbed metabolic pathways, providing a visual overview of metabolic changes associated with cerebral infarction, thrombosis, and recovery (Figure 5).

Pathway enrichment analysis revealed that six signaling pathways were elevated in FTR subjects compared with healthy controls (Figure 5A). Among these, oxidative phosphorylation (OXPHOS)‐related pathways were prominently enriched. OXPHOS is central to mitochondrial ATP production, and platelets utilize both OXPHOS and glycolysis to meet their energy demands. Prior studies have shown that impaired electron transport chain activity can increase mitochondrial reactive oxygen species (mtROS) [25, 26], which have been associated with platelet activation, neutrophil extracellular trap (NET) formation, and endothelial dysfunction [27, 28]. The enrichment observed here is therefore consistent with previously reported links between mitochondrial metabolism and thrombotic processes in cerebral infarction; however, mitochondrial function and ROS levels were not directly measured in this study. Accordingly, these findings should be interpreted as associative and hypothesis‐generating rather than evidence of active mitochondrial dysfunction in this cohort.

The enrichment of pathways related to parathyroid hormone (PTH) signaling may reflect broader endocrine–vascular interactions. PTH is a key regulator of calcium and phosphate homeostasis, and elevated PTH levels have been associated with endothelial dysfunction, vascular remodeling, and inflammatory signaling in prior studies [29]. While these observations suggest potential links between PTH‐related pathways and vascular pathology, circulating hormone levels and downstream functional effects were not assessed in the present study. Therefore, the observed pathway enrichment should be interpreted cautiously as an association rather than evidence of a causal endocrine contribution to thrombosis in cerebral infarction.

One enriched KEGG pathway was annotated as Parkinson's disease (PD). This finding should not be interpreted as indicating the presence of PD in the study population. Rather, the KEGG PD pathway captures shared biological features such as mitochondrial respiratory chain dysfunction, oxidative stress, and altered energy metabolism. Its enrichment in this context likely reflects overlap with these general processes rather than disease‐specific pathology. While such processes have been implicated in vascular and platelet biology relevant to cerebral infarction, they were not directly evaluated in the present study.

In addition to the pathways described above, sphingolipid metabolism, sphingolipid signaling, and central carbon metabolism in cancer were enriched in FTR patients and remained elevated in the MFR group relative to healthy controls, suggesting incomplete normalization following recovery. Sphingolipids are a class of bioactive lipids with structural and signaling roles in mammalian cells [30, 31], and specific species, including ceramides and sphingosine‐1‐phosphate (S1P), have been implicated in endothelial function, platelet activity, and coagulation in prior studies [32, 33, 34, 35, 36]. Importantly, sphingolipid signaling is highly context‐dependent, as different species can exert distinct, and sometimes opposing, biological effects [37, 38]. The enrichment of sphingolipid‐related pathways in both FTR and MFR groups may therefore reflect a shared metabolic response to thrombotic injury and/or thrombectomy for cerebral infarction, whereas the differences observed between these groups could indicate variations in the relative abundance or composition of specific sphingolipid species rather than uniform pathway activation. Such differences may be consistent with shifts in sphingolipid metabolic balance reported in prior studies; however, the present analysis does not resolve individual lipid species or metabolic flux in sufficient detail to define these changes. Accordingly, the observed differences between MFR and FTR should be interpreted as group‐level metabolic distinctions that may reflect underlying biological heterogeneity, rather than direct evidence of specific regulatory mechanisms.

Central carbon metabolism pathways represent core processes involved in energy production and biosynthesis [39] and are commonly identified in pathway enrichment analyses due to their broad involvement in cellular metabolism. Although alterations in these pathways have been associated with changes in cellular microenvironments, including metabolite accumulation and pH shifts, and have been linked to vascular and immune function in prior studies [40, 41], the current data do not directly assess these processes. Therefore, the enrichment of central carbon metabolism pathways should be interpreted as indicative of altered metabolic states rather than evidence of specific downstream functional effects in the context of cerebral infarction recovery.

Although these pathways have been individually linked to thrombotic and inflammatory processes associated with cerebral infarction, the present analysis is based on pathway enrichment and does not directly measure metabolite flux, signaling activity, or functional interactions among these systems. Therefore, the observed co‐enrichment should not be interpreted as evidence of an integrated mechanistic network, but rather as a set of related metabolic alterations that may reflect underlying biological states associated with cerebral infarction and recovery. These observations may help generate testable hypotheses for future studies.

Multiple altered metabolic pathways were identified in MFR subjects compared with both healthy controls and FTR patients (Figure 5B,C), including pathways related to inflammatory processes, neurotransmission, hormonal signaling, proteolysis, mineral metabolism, glucose utilization, amino acid metabolism, and lipid metabolism. Many of these pathways have been implicated in mechanisms relevant to thrombosis, such as endothelial dysfunction, platelet activation, and inflammatory signaling, especially in the context of post‐cerebral infarction recovery. Consistent with prior literature, metabolic reprogramming in vascular and immune cells has been associated with prothrombotic phenotypes [42, 43]. However, the present study does not directly assess these cellular processes and therefore cannot establish mechanistic links between the observed metabolic alterations and specific thrombotic pathways. These findings should be interpreted as providing a framework for hypothesis generation rather than mechanistic inference.

These observations raise the possibility that sphingolipid‐related and central metabolic pathways may represent candidate areas for further investigation in the context of cerebral infarction recovery. Future studies could evaluate whether specific metabolites or pathway signatures have utility as biomarkers of thrombotic risk or recovery after cerebral infarction, and whether modulation of these pathways influences vascular or platelet function. However, such applications remain speculative and require validation in independent cohorts as well as functional characterization in experimental systems.

This study provides a comprehensive metabolomic characterization of patients with distinct clinical outcomes following thrombectomy for cerebral infarction and identifies outcome‐associated metabolic differences that may offer insight into thrombotic biology. By integrating multivariate and univariate analytical approaches, the study highlights biologically relevant pathways potentially linked to vascular and platelet function in cerebral infarction, while maintaining an association‐based interpretive framework.

Several limitations of this study should be acknowledged. First, metabolomic profiling was performed at a single time point, which precludes assessment of temporal dynamics and limits insight into how metabolic changes evolve during thrombosis and post‐thrombectomy recovery in cerebral infarction. Second, the study lacks independent external validation and functional experiments, and therefore, the observed metabolite alterations should be interpreted as associative rather than causal. Third, although modest, a statistically significant age difference was observed between the MFR and FTR groups, which may introduce potential confounding effects. Fourth, the relatively small sample size may limit statistical power and the generalizability of the findings.

Future studies are needed to address these limitations. Longitudinal sampling designs will be important to characterize dynamic metabolic trajectories over time after cerebral infarction. Independent validation in larger, well‐characterized cohorts with balanced clinical variables is required to confirm the robustness of the identified metabolic patterns. In addition, mechanistic studies, such as targeted metabolite perturbation, in vitro platelet and endothelial assays, and in vivo models, will be necessary to establish causal links between specific metabolic pathways and thrombotic or recovery processes in cerebral infarction. Together, these efforts will be essential to determine the biological significance and evaluate the translational relevance of the metabolomic findings.

In summary, our untargeted metabolomics analysis identified broad metabolic differences across the CON, FTR, and MFR groups, highlighting pathways that may be associated with thrombosis and recovery after cerebral infarction. Several findings are consistent with prior reports, supporting their potential biological relevance; however, their clinical utility remains to be established. Future work should validate these candidate metabolites and pathways in larger, independent cohorts and assess their robustness, specificity, and relationship to clinical outcomes before considering potential applications in diagnosis, monitoring, or risk stratification for patients with cerebral infarction.

## Author Contributions

K.C., Y.M., Y.X., and S.F. conceived the study. M.C., X.H., and S.D. were responsible for patient recruitment, clinical evaluation, and sample and data acquisition. W.S., D.G., and X.Z. performed data curation and statistical analyses. M.C. and X.H. contributed to data interpretation. Y.X. drafted the initial manuscript, K.C., Y.M., and S.F. critically revised the manuscript for important intellectual content. K.C. and Y.M. contributed to project administration and oversight. All authors reviewed and approved the final manuscript and agree to be accountable for all aspects of the work.

## Funding

This work was supported by the Chongqing Outstanding Young Medical Talent Award (YXGD202503) and the Hospital Talent Program (YJJL2025024, YJJL2025003).

## Ethics Statement

All study procedures were reviewed and approved by the institutional ethics committee (Approval No. 2023LLS027), and written informed consent was obtained from each participant prior to enrollment.

## Conflicts of Interest

The authors declare no conflicts of interest.

## Supporting information


**Table S1:** Source data for pairwise OPLS‐DA score plots showing separation between groups.

**Table S2:** Source data for heatmap analysis illustrating expression patterns of differentially expressed metabolites across CON, FTR, and MFR groups.

**Table S3:** Source data for volcano plot analyses for all pairwise group comparisons.

**Table S4:** Source data for significantly enriched metabolic pathways identified in each pairwise comparison.

## Data Availability

The data that support the findings of this study are available from the corresponding author upon reasonable request.
